# Extracorporeal Membrane Oxygenation Supported Transbronchial Cryobiopsy in the Diagnosis of Severe Organizing Pneumonia: A Case Report

**DOI:** 10.3389/fmed.2022.955992

**Published:** 2022-07-14

**Authors:** Xiaoyan Zhang, Yuqiong Wang, Yingying Feng, Ling Zhao, Yunxia Zhang, Hanbo Yang, Bin Xing, Wenlin Guo, Ting Sun, Qingyuan Zhan, Ye Tian

**Affiliations:** ^1^Peking University China-Japan Friendship School of Clinical Medicine, Beijing, China; ^2^Department of Pulmonary and Critical Care Medicine, Center of Respiratory Medicine, National Center for Respiratory Medicine, China-Japan Friendship Hospital, Beijing China; ^3^Department of Pathology, China-Japan Friendship Hospital, Beijing, China; ^4^Department of Rheumatology, China-Japan Friendship Hospital, Beijing, China; ^5^China-Japan Union Hospital of Jilin University, Changchun, China

**Keywords:** organizing pneumonia, extracorporeal membrane oxygenation, mechanical ventilation, transbronchial cryobiopsy, case report

## Abstract

This case report describes a 58-year-old, never-smoking housewife with chief complaints of progressively worsening cough, dyspnea, and intermittent fever, who was initially misdiagnosed with community-acquired pneumonia (CAP). However, her pulse oximetry oxygen saturation continued to decline, and eventually, she underwent an endotracheal intubation. Fortunately, transbronchial cryobiopsy (TBCB) assisted by extracorporeal membrane oxygenation (ECMO) was performed in the most critical situation, and it revealed an organizing pneumonia (OP) pattern. OP describes a histological pattern of acute or subacute pulmonary damage, which may be idiopathic or associated with a known or unknown underlying disease. A definitive diagnosis of OP usually obtained from pathology, and surgical lung biopsy with large lung tissue is recommended. However, since the surgical lung biopsy was not convenient for this patient after mechanical ventilation, bedside TBCB supported by ECMO was selected. To our knowledge, we are the first to report the pathological diagnosis of ECMO assisted TBCB in acute respiratory failure. When oxygenation cannot be maintained after endotracheal intubation and surgical lung biopsy is not feasible, ECMO-supported TBCB may be a good choice to obtain lung tissue for histopathological diagnosis in patients with acute lung injury of unknown etiology.

## Introduction

Organizing pneumonia (OP) describes a histological pattern of acute or subacute pulmonary damage. The first description of OP can be traced back to Lange in 1901 (1). It is a clinical entity (1–3) associated with non-specific clinical manifestations, and is associated with a known or unknown underlying disease, leading to delay in diagnosis, or even misdiagnosis. A definitive diagnosis is usually obtained from lung biopsy because of the unavailability of other specific detection methodologies (4). Corticosteroid treatment represents the standard therapy in OP (5).

In this report, we present the case of a patient who was initially misdiagnosed with community-acquired pneumonia (CAP), and her respiratory failure progressed rapidly. Fortunately, transbronchial cryobiopsy (TBCB), assisted by extracorporeal membrane oxygenation (ECMO), was performed in the most critical situation, and it revealed an OP pattern. TBCB supported by ECMO has improved the pathological diagnosis rate of unexplained acute respiratory failure to a certain extent, which is significant in guiding precise treatment and disease prognosis. This study aims to suggest an alternative approach to the diagnosis of OP in the scenario of an acute respiratory failure.

## Case Presentation

**FIGURE 1:**
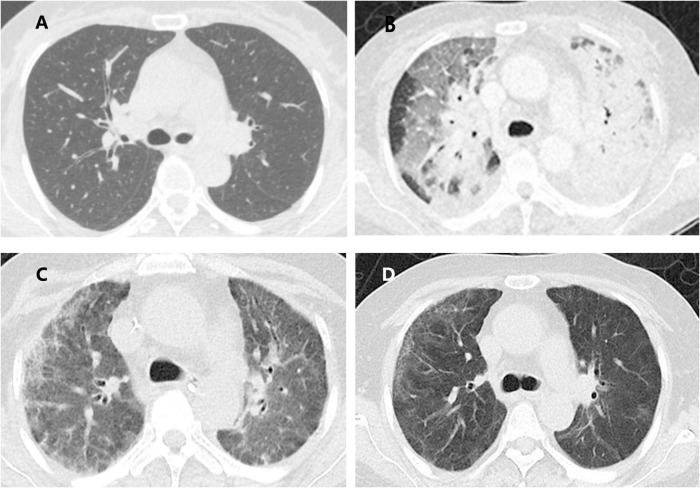
Evolution of the computed tomography (CT) findings of the patient. **(A)** Scattered solid nodules in both lungs, with fibrous stripe shadow in the medial middle lobe of the right lung and slight enlargement in the left hilum; **(B)** patchy areas of ground glass opacities (GGO), consolidation in the double lungs scattered all over both lungs and slightly enlarged mediastinal lymph nodes; **(C)** consolidation and GGO in both lungs decreased compared with the previous one; **(D)** only a small amount of GGO and fibrous stripe shadow.

A 58-year-old, never-smoking housewife presented to the Department of Pulmonary and Critical Care Medicine of China-Japan Friendship Hospital with the chief complaints of cough, dyspnea, and intermittent fever. She had a history of leukopenia of unknown origin that was untreated. Chest computed tomography (CT) revealed scattered solid nodules in both lungs, with a fibrous striped shadow in the medial segment of the middle lobe of the right lung and slight enlargement in the left hilum (Figure 1A). Though broad-spectrum antibiotics were administered, the patient’s clinical status worsened, developing a severe pneumonia with type I respiratory failure and her pulse oximetry oxygen saturation (SpO_2_) decreased to 70% while on oxygen delivered at the rate of 5 L/min through a nasal cannulae. The patient was transferred to the intensive care unit (ICU) for close monitoring.

A general physical examination was performed. The patient, who was conscious, displayed the following vital signs: temperature, 38.6°C; pulse rate, 105 bpm; respiratory rate, 35 breaths/min; blood pressure, 118/77 mmHg; oxygen saturation, 93% while on oxygen delivered through a nasal cannulae at a rate of 5 L/min. Chest auscultation revealed raw breathing sounds and wet rales in both lungs. Pitting edema in both lower extremities was found as well. Results of laboratory tests were: white blood cell count 8.54*10^9/L with neutrophil predominance (89.7%); reactive protein > 200mg/L (reference value <10 mg/L); serum procalcitonin 1.67 ng/mL, alanine transaminase 48 U/L, aspartate transaminase 68 U/L. Autoimmune tests including anti-neutrophil antibody (ANA), anti-neutrophil cytoplasmic antibody (ANCA), and rheumatoid factor (RF) were negative. Results of blood culture, 1-3-β-D-glucan (BDG) assay, galactomannan (GM) tests, T-spot, cryptococcal antigen nucleic acid, blood EB virus DNA, cytomegalovirus DNA were negative. Fiberoptic bronchoscopy with bronchoalveolar lavage (BALF) revealed extensive hyperemia and edema in the bronchial mucosa, and no other abnormalities were observed. Fungal cultures were positive for *Candida albicans*. Cultures for bacteria and mycobacteria were negative. All virus, *Mycoplasma pneumoniae*, and Chlamydia nucleic acid amplification tests were negative. No pneumocystis infection was identified and tumor cytology was negative. Contrast-enhanced chest CT revealed patchy areas of ground-glass opacities (GGO) and consolidation in both lungs scattered all over and with slightly enlarged mediastinal lymph nodes (Figure 1B).

**FIGURE 2:**
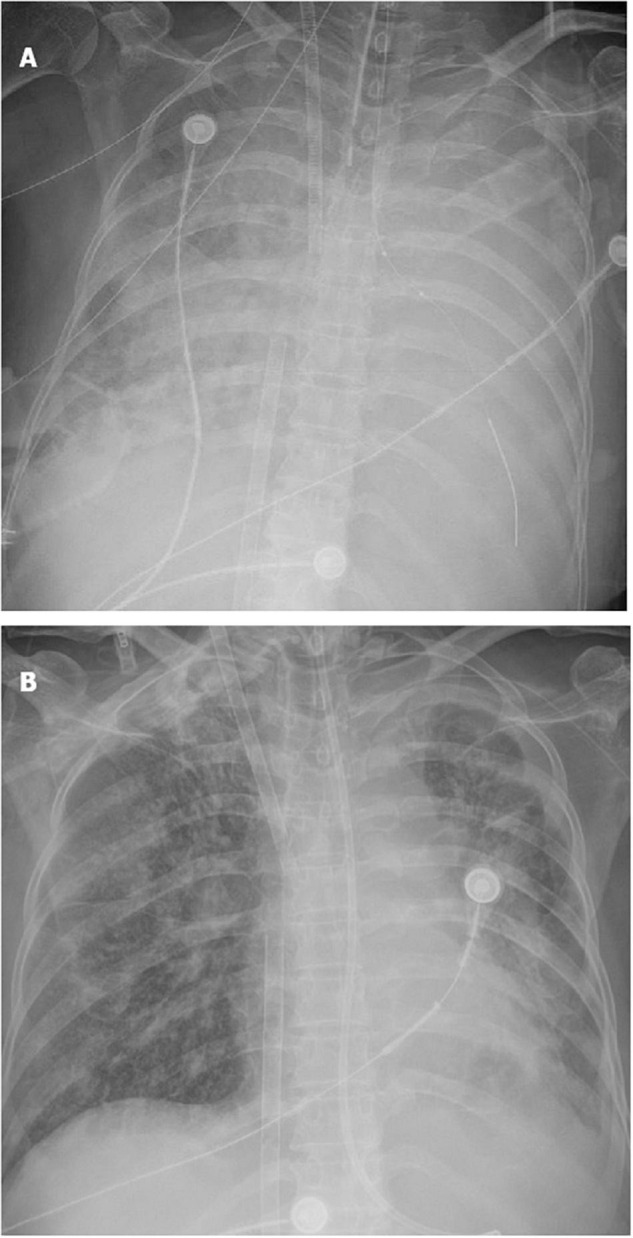
Evolution of the chest X-ray of the patient. **(A)** Extensive consolidation of both lungs; **(B)** consolidation of both lungs decreased significantly.

**FIGURE 3:**
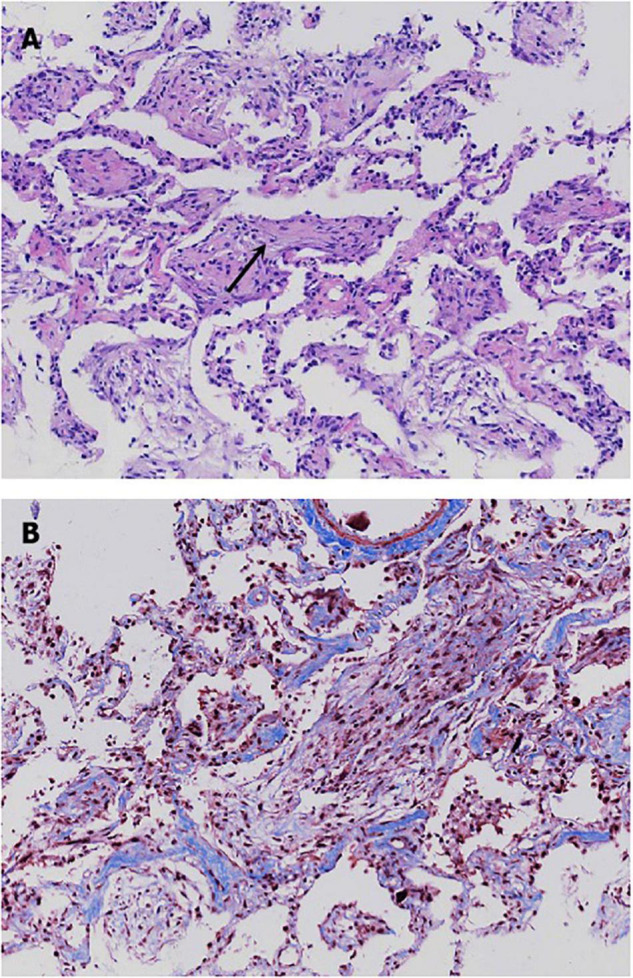
Histological patterns of the patient’s lung tissue. **(A)** Hematoxylin-eosin stain revealed the presence of fibroblasts intermixed with loose connective tissue and collagen in the alveoli and the distal bronchioles. **(B)** Masson stain showed a mass of blue collagen fibers stained with aniline blue.

On ICU day 2, her respiratory status continued to decline, and bedside chest radiographs (Figure 2A) showed progression of bilateral infiltrates. Mechanical ventilation (PC mode, FiO_2_ 100%, F 35/min, PC 18 cmH_2_O, PEEP 10 cmH_2_O), and venovenous extracorporeal membrane oxygenation (V-V ECMO) were performed to maintain adequate oxyhemoglobin saturation. We delivered an almost constant flow of 6 L/min/m^2^ *via* ECMO. A bedside TBCB was performed on the same day to obtain a definitive diagnosis. Three specimens were obtained from the outer and posterior basal segments of the lower lobe of the left lung, and histological examination revealed fibroblasts intermixed with loose connective tissue and collagen in the alveoli and the distal bronchioles (Figures 3A,B). These findings were typical of OP (5). Thus, an OP diagnosis was established.

The following day, the patient’s treatment was changed to intravenous methylprednisolone in continuous infusion, 240 mg for 3 days, followed by 80 mg for 5 days, and then 40 mg as the maintenance dose. The fever subsided the day after the steroid was given, and her general condition improved significantly. The patient was extubated 4 days after endotracheal intubation and tracheotomized for 24 days. She remained on VV-ECMO for 15 days. Systemic heparinization is required during ECMO treatment. On the one hand, thrombus formation can be seen in front of the ECMO membrane and behind the membrane, and thrombosis can be seen around the PICC tube. On the other hand, the patient developed hemoptysis several times, hematuria, hematoma of abdominal muscle successively. For hemoptysis, we did a bronchoscopy to stop the bleeding. And we adjusted the heparin pump rate according to the APTT target value to prevent massive bleeding. After the patient was withdrawn from ECMO, heparin was stopped, and the hemoptysis was gradually relieved. There was no recurrence of hematuria and no increase in abdominal hematoma. No thrombosis was found in the deep veins of the extremities.

Bedside chest radiographs showed consolidation of both lungs decreased significantly on ICU day 10 (Figure 2B). On day 21 after steroid use, her chest radiograph (Figure 1C) showed that the consolidation was less than the previous. On day 41 after steroid use, her symptoms and chest radiograph (Figure 1D) were indicative of a significant improvement, with complete resolution of pulmonary symptoms.

## Discussion

To our knowledge, ECMO-assisted TBCB has never been reported in the pathological diagnosis of acute respiratory failure.

Clinically, patients with OP may have acute or subacute disease progression that may include constitutional symptoms with flu-like illness, including cough, fever, malaise, weight loss, and dyspnea (1, 6). OP may be idiopathic or associated with a known or unknown underlying disease, leading to delay in diagnosis or even misdiagnosis. Similarly, our patient was diagnosed with infectious pneumonia initially and was treated with antibiotics, which may have delayed her early steroid use and led to the eventual progression of her illness. Fortunately, the patient in our case recovered, which may be because ECMO-supported TBCB was decisively performed on this patient, enabling us to develop a pathological diagnosis and use glucocorticoids at the right time.

Obtaining lung tissue for histopathological diagnosis in patients with acute lung injury of unknown etiology is helpful for precision therapy. This is because of the atypical symptoms, non-specific laboratory findings, and variable CT findings associated with OP (7). The patient had undergone ultrasound-guided percutaneous transthoracic needle biopsy in the general ward, but no valuable results were obtained. The failure of the aforementioned procedure was mainly due to the respiratory distress, which made impossible for the patient to hold her breath, resulting in unsatisfactory lung tissue sampling. A study (8) pointed out that it is difficult to diagnose OP based on bronchoscopic biopsy specimens because of its patchy distribution. Methods of obtaining large specimens, such as surgical lung biopsy, are better options for the disease. However, in our case, oxygenation could not be maintained after endotracheal intubation, making surgical lung biopsy even more challenging to perform. In our case, ECMO rendered possible a safe bedside TBCB in an otherwise extremely unstable patient.

Currently, VV-ECMO has been described for managing different lung diseases in the ICU for severe cardiorespiratory failure. However, ECMO-assisted TBCB is rarely reported in the pathological diagnosis of acute respiratory failure. Our patient’s deteriorating clinical condition was a major limitation of lung biopsy. Thus, she underwent TBCB with VV-ECMO support as a bridge to diagnosis and treatment. This may show that ECMO-assisted TBCB is feasible for the histopathological diagnosis of acute respiratory failure of unknown cause. ECMO enables biopsy in critically ill patients to find the exact pathological evidence in acute lung injury management.

OP is a unique model of intraalveolar fibrotic inflammation that is fully reversible with treatment (5). However, the disease continued to progress in this case despite receiving 40 mg/day of methylprednisolone outside our hospital for 9 days, perhaps due to the inadequate treatment of steroids. The day after the patient was diagnosed, we administered pulse corticosteroid treatment of methylprednisolone 240 mg/day in continuous infusion for 3 consecutive days and then reduced it to 80 mg/day. We believe that the patient’s improvement is largely dependent on appropriate steroid therapy. The standard treatment for OP has not yet been determined (9–11). For patients with cryptogenic OP, the recommended initial prednisone dose is 0.75 mg/kg/day for 2–4 weeks (12). However, physicians still need to adjust the dose of prednisone based on disease severity and response to therapy. In severe OP, intravenous prednisolone can be administered for 3 consecutive days (13–15) and immunosuppressive therapy with cyclophosphamide, azathioprine, or cyclosporin A can be used (16, 17), especially for acutely ill patients.

In addition, it is important to find the right opportunity to use ECMO and avoid the associated complications. Bleeding and thrombosis are serious complications of ECMO. Dealing with possible complications requires continuous management from relevant experts. One reason for the successful treatment of this patient is that we used ECMO at the right time and cleverly managed its complications.

## Conclusion

OP is an unusual cause of acute lung injury. Diagnosis is mostly biopsy-proven. When oxygenation cannot be maintained after endotracheal intubation and surgical lung biopsy is not feasible, ECMO-supported TBCB may be a good choice to obtain lung tissue for histopathological diagnosis in patients with acute lung injury of unknown etiology, because ECMO can provide a relatively safe bedside TBCB for extremely unstable patients to avoid delayed diagnosis or even misdiagnosis. However, care should be taken to avoid ECMO-related complications.

## Data Availability Statement

All data discussed in this manuscript are included within this published article.

## Ethics Statement

Ethical review and approval were not required for the study on human participants in accordance with the local legislation and institutional requirements. The patient provided her written informed consent to participate in this study. Written informed consent was obtained from the individual for the publication of any potentially identifiable images or data included in this article.

## Author Contributions

YW, XZ, YF, and LZ produced the first draft of the manuscript. YZ, HY, BX, WG, and TS were major contributors in analyzed and interpreted the patient’s data. YT, XZ, and QZ were major contributors in writing the manuscript. All authors read, approved the final manuscript, reviewed, edited, and approved the final versions of the submitted manuscript.

## Conflict of Interest

The authors declare that the research was conducted in the absence of any commercial or financial relationships that could be construed as a potential conflict of interest.

## Publisher’s Note

All claims expressed in this article are solely those of the authors and do not necessarily represent those of their affiliated organizations, or those of the publisher, the editors and the reviewers. Any product that may be evaluated in this article, or claim that may be made by its manufacturer, is not guaranteed or endorsed by the publisher.
